# 2620. Normalization of eosinophil count is predictive of reduced oxygen requirement during Covid-19 infection

**DOI:** 10.1093/ofid/ofad500.2233

**Published:** 2023-11-27

**Authors:** Benjamin Davido, Karim Jaffal, Isabelle Vaugier, Stephane Bourlet, Pierre De Truchis, Djillali Annane

**Affiliations:** Raymond Poincaré Teaching Hospital, Garches, Ile-de- France, France; Hopital Raymond Poincaré, Garches, Ile-de-France, France; Hopital Raymond Poincaré, Garches, Ile-de-France, France; Hopital Raymond Poincaré, Garches, Ile-de-France, France; Hopital Raymond Poincaré, Garches, Ile-de-France, France; Hopital Raymond Poincaré, Garches, Ile-de-France, France

## Abstract

**Background:**

The initial need for oxygen during Covid-19 has been correlated with the severity of lung involvement observed on CT-scan caused by the cytokine storm. A recent study showed that the normalization of eosinophil count (≥100/mm^3^) after 48 hours of appropriate regimen was related to a favorable outcome in sepsis. Our work intends to assess the kinetics of eosinophil count over time based on oxygen levels during the acute phase of this viral infection.

**Methods:**

Retrospective study, conducted between March-April 2020 (first wave), among adults admitted directly to a medicine ward. Biological abnormalities, including lymphocyte count, eosinophil count, and CRP were gathered daily during the first week of admission (day 0) according to oxygen level. In case of worsening requiring ICU, oxygen level was censored at 15 L/min. Aim of the study was to investigate whether normalization of eosinophil count could predict a decrease in oxygen requirements, and potentially patient’s outcome. Values were presented as median with 95% CI and statistical analysis utilized the Chi-2 square test.

**Results:**

132 patients were admitted, mean age 59.0±16.3 years. Mean onset of symptoms was 7±4 days. Majority of patients had cardiovascular disease (43.1%), underlying respiratory disease (16.7%), diabetes (18.9%), immunosuppression (8.3%) and obesity (10.6%).

At admission, median (IQR) CRP=79(26-130) mg/L, lymphocyte count=1005(730-1380)/mm^3^ and eosinophil count=10(0-60)/mm^3^. 72% of patients required oxygen supply. Lung CT-scan (n=103) revealed: 53.5% had moderate (25-50%) to severe ( >50%) involvement.

Twenty-seven patients (20.5%) required ICU due to condition worsening.

Eosinophil count increased continuously until normalization from day0 to day3, while the oxygen level started to decrease on day 3 without any rebound observed thereafter (Figure 1). Similarly, CRP decreased to 43 mg/L on day2, but ultimately increased again. Patients requiring < 2L/min of oxygen were significantly associated with an eosinophil count≥100/mm^3^ on day 2 OR=0.2 [0.07-0.56] (p=0.002).

**Figure:**
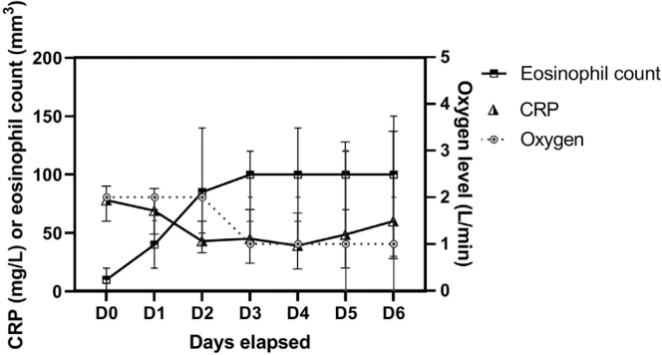

**Conclusion:**

Normalization of eosinophil count over time seems predictive of a decrease of oxygen requirement during Covid-19. Those data should be confirmed on other type of respiratory tract infections.

**Disclosures:**

**All Authors**: No reported disclosures

